# The utilization of a novel Outpatient Appropriateness Fragility Score to predict inpatient stay following biportal lumbar endoscopic decompression

**DOI:** 10.1016/j.xnsj.2025.100752

**Published:** 2025-06-18

**Authors:** Thomas E. Olson, Carlos Maturana, Christopher D. Hamad, Alex M. Upfill-Brown, William L. Sheppard, Don Young Park

**Affiliations:** a Department of Orthopaedic Surgery, David Geffen School of Medicine at UCLA, 1250 16th St., Santa Monica, CA 90404, United States; b Department of Orthopaedic Surgery, UC Irvine School of Medicine, 101 The City Drive South, Pavillion III, Building 29A, Orange, CA 92868, United States

**Keywords:** Outpatient surgery, Lumbar decompression, Fragility, Frailty, Minimally invasive spine surgery (MISS), Biportal endoscopic spine surgery

## Abstract

**Background:**

Biportal endoscopic spine surgery offers advantages such as reduced postoperative pain and faster recovery, often enabling same-day discharge. However, the patient-specific factors influencing the need for inpatient admission remain unclear. This study evaluates variables contributing to overnight stays following biportal lumbar endoscopic decompression and proposes a predictive fragility score.

**Methods:**

A retrospective analysis of prospectively collected data was conducted on 84 consecutive patients undergoing one- or two-level lumbar endoscopic decompression at a single U.S. academic center. Patients with trauma, tumor, infection, or revision procedures were excluded. Cohorts were divided by discharge status: same-day discharge (outpatient) versus one or more night hospital stay (inpatient). A novel fragility score (4–21 points) incorporating age, body mass index (BMI), comorbidities, and procedure type was developed. Sarcopenia was assessed using the psoas muscle index (PMI), defined as the ratio of psoas to vertebral cross-sectional area on preoperative imaging. Cutoff values were analyzed via Youden’s *J* statistic and receiver operating characteristic analysis.

**Results:**

Same-day discharge patients were significantly younger (55.3 vs. 68.5 years; p=.0003) and had lower American Society of Anesthesiologists (2.0 vs. 2.7; p<.0001) and Charlson Comorbidity Index scores (1.6 vs. 3.5; p<.0001). No significant BMI difference was observed (p=.4341). Outpatients more frequently underwent discectomy; inpatients more commonly received ULBD and two-level decompression (p<.0001, p=.0014). A fragility score ≥11 predicted inpatient stay with an area under the curve (AUC) of 0.810, outperforming Modified 5-Item Frailty Index (AUC 0.640). PMI did not differ between groups (p=.6732), with AUCs of 0.417 overall, and 0.482 (males), 0.487 (females). Fragility score and PMI were weakly correlated (*r*=–0.130).

**Conclusions:**

The proposed Outpatient Appropriateness Fragility Score effectively predicts inpatient admission after biportal lumbar decompression. Factors such as age, comorbidities, and surgical extent are more predictive than BMI or sarcopenia. This tool may guide preoperative planning and optimize resource utilization.

## Introduction

The global population is aging, and with this demographic shift comes an increase in age-related comorbidities, including cardiovascular disease, diabetes, and decreased muscle mass [1,2]. As individuals live longer, the burden of degenerative conditions, particularly those affecting the spine, continues to grow. Degenerative spine disorders, such as lumbar disc herniation and spinal stenosis, often lead to debilitating pain, reduced mobility, and decreased quality of life particularly with increasing age. These conditions frequently necessitate surgical intervention, yet the presence of comorbidities in this population can complicate perioperative management and elevate the risk of postoperative complications [[3], [4], [5]].

Spine surgery in older, often frail, patients is associated with considerable risks, including prolonged hospital stays, complications, and even mortality. Frailty—characterized by diminished physiological reserves and increased susceptibility to stressors—has been identified as a key predictor of poor postoperative outcomes in spinal surgery [[6], [7], [8], [9], [10]]. In recent years, research has shown that higher frailty scores correlate with extended inpatient stays and an increased likelihood of adverse events, underscoring the need for accurate preoperative risk assessment in this vulnerable population [11,12].

Minimally invasive spine surgery (MISS) has emerged as a potential solution to some of these challenges, particularly in the treatment of lumbar degenerative conditions [[13], [14], [15]]. By reducing soft tissue disruption and minimizing blood loss, MISS techniques have been shown to lessen recovery times and improve postoperative outcomes compared to traditional open surgeries. However, even with the benefits of MISS, older patients with complex health profiles may still face significant postoperative risks.

**Fig. 1:**
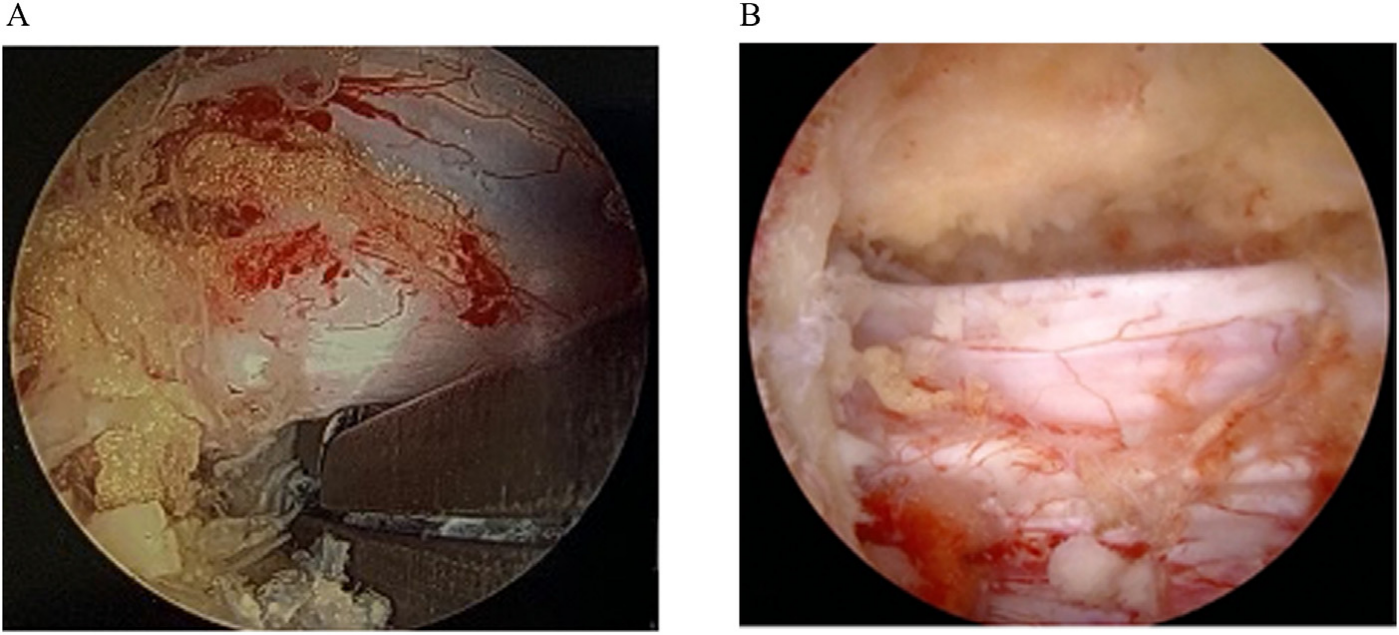
*Intraoperative and endoscopic images illustrating biportal endoscopic lumbar decompression*. (A) Endoscopic view of a pituitary rongeur adjacent to the traversing nerve root in the disc space during an endoscopic discectomy. (B) Endoscopic view of the thecal sac decompressed bilaterally and dorsally during an endoscopic unilateral laminotomy, bilateral decompression for lumbar stenosis.

Endoscopic surgery, a growing MISS technique, has gained popularity for lumbar decompression due to its ability to achieve effective decompression with minimal tissue disruption [16,17]. Biportal endoscopic decompression (Fig. 1), for instance, offers a targeted approach to address lumbar spine pathologies while potentially reducing the need for extensive hospital stays [18]. Yet, patient outcomes after endoscopic decompression remain variable, especially in populations with medical comorbidities associated with higher fragility scores [[19], [20], [21], [22]]. In light of this, understanding which patient-specific factors contribute to prolonged hospitalization can inform preoperative planning and improve patient care. The Modified 5-Item Frailty Index (mFI-5) has emerged as a commonly used tool among spine surgeons due to it’s association with greater morbidity and mortality in spine surgery, however, studies demonstrating this often focus on procedures with greater baseline risk and expected inpatient stays, including trauma, fusion, and deformity correction [11,[23], [24], [25]].

This study seeks to address this knowledge gap by proposing a novel Outpatient Appropriateness Fragility Score (OAFS) tailored to patients undergoing biportal lumbar endoscopic decompression. Through retrospective analysis, we examine demographic, clinical, and surgical variables—including age, comorbidities, and sarcopenia quantified by the psoas muscle index (PMI)—to determine which factors predict the need for inpatient recovery. By identifying predictors of prolonged stays, this novel scoring system aims to enhance risk stratification and optimize resource allocation in managing patients undergoing minimally invasive lumbar spine surgery.

## Methods

### Study design and patient selection

Consecutive patients undergoing biportal spinal endoscopy by a single surgeon at a tertiary care university hospital system in the United States were included in this study. The study was prospectively collected from October 2021 through February 2023 and retrospectively analyzed under UCLA IRB#18-000760. Inclusion criteria were patients with a preoperative indication of lumbar disc herniation and/or central canal stenosis with complete perioperative data and minimum of 90-day follow-up. Exclusion criteria included cases involving trauma, tumor, infection, and revision surgery. Patients were retrospectively categorized into two cohorts: those discharged on the same day of surgery (outpatient) and those who required one or more nights of overnight observation or hospital admission (inpatient). All patients were required to clear standards of mobility, function, and pain thresholds set by the health system including multidisciplinary input prior to the final discharge decision made by the operating surgeon.

### Data collection and variables

**Fig. 2:**
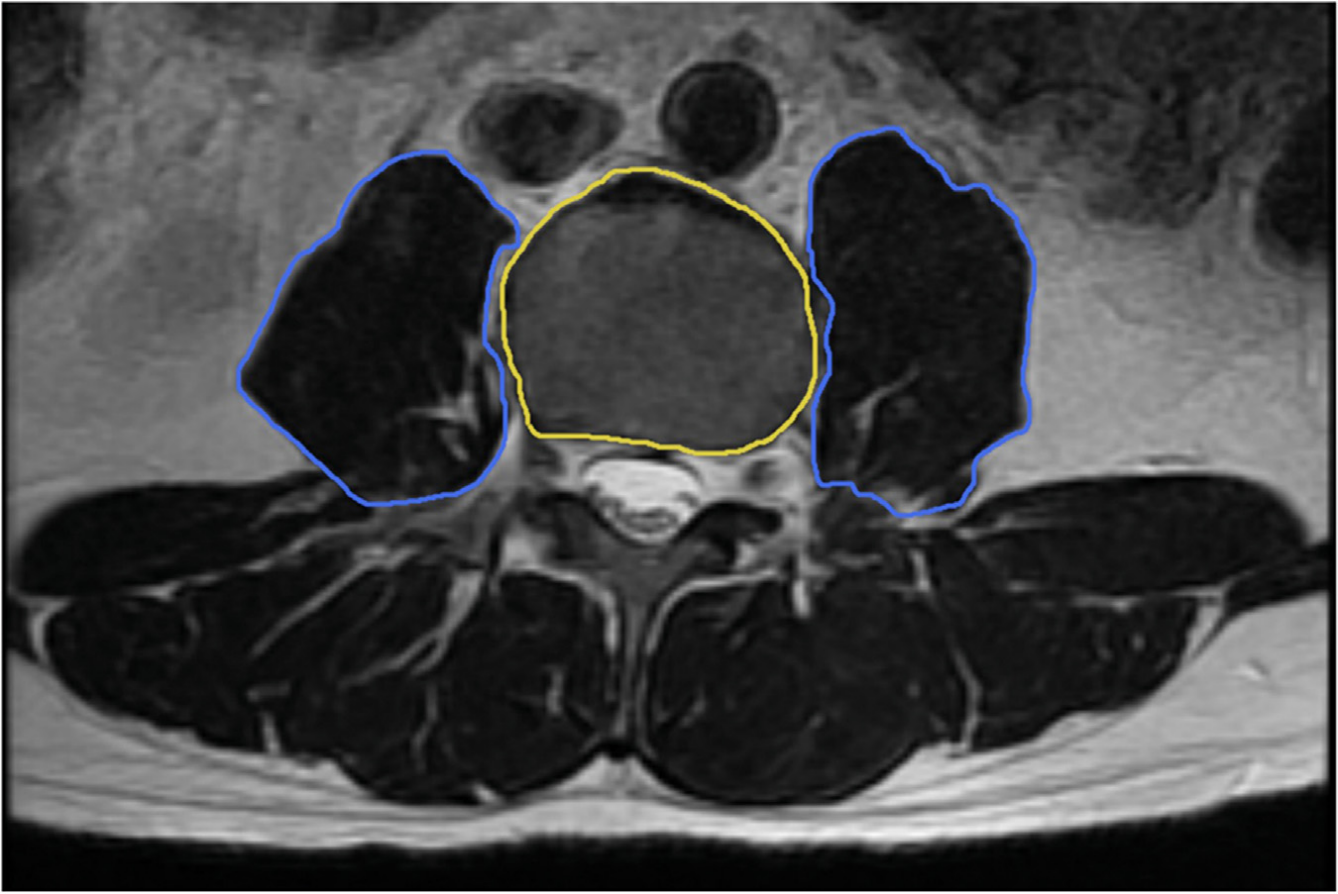
*Representative T2-weighted axial MRI at the level of the third lumbar vertebral body (L3) illustrating the method for calculating the Psoas Muscle Index (PMI)*. The bilateral psoas muscles are highlighted in blue to delineate their cross-sectional areas (CSA), and the L3 vertebral body is highlighted in yellow. The PMI is calculated by dividing the combined CSA of bilateral psoas muscles by the CSA of the L3 vertebral body.

Demographic and clinical variables collected included age, sex, body mass index (BMI), American Society of Anesthesiologists (ASA) score, Charlson Comorbidity Index, and the specific surgical procedure performed. Operative data such as surgical duration, estimated blood loss, drain utilization and duration, and postoperative complications were recorded. The OAFS was calculated for each patient using a novel proposed scoring system, which accounts for age, BMI, procedure type, number of levels, and comorbidities, with scores ranging from 4 to 21 points (Supplementary Table 1) that includes the mFI-5 [11]. Sarcopenia was evaluated using the PMI, calculated as the ratio of the cross-sectional area of the psoas muscle to bone cross-sectional area in preoperative imaging (Fig. 2), as reported previously by Kong et al [26].

### Outcome measures

The primary outcome was the requirement of an inpatient stay following the procedure. Secondary outcomes included operative variables such as surgical duration, drain output, and postoperative complications.

### Statistical analysis

Comparative statistics were used to analyze differences between the outpatient and inpatient groups. Continuous variables were analyzed using *t* tests, while categorical variables were analyzed using chi-square or Fisher’s exact test as appropriate. The predictive ability of the OAFS and mFI-5 scores for inpatient stay were assessed using receiver operating characteristic analysis, with Youden’s *J* statistic employed to determine optimal cutoff values. Correlations between the OAFS score, mFI-5, and PMI were evaluated using Pearson’s correlation coefficient.

## Results

### Patient demographics and baseline characteristics

A total of 84 patients were included, with 54 (64%) undergoing outpatient surgery and 30 (36%) requiring an inpatient stay. The demographic characteristics are outlined in Table 1. Patients in the outpatient cohort were significantly younger than those in the inpatient group (mean age 55.3 vs. 68.5 years, p=.0003). There was no significant difference in BMI between the two groups (mean BMI: 27.3 vs. 28.1, p=.4341). However, inpatients exhibited higher ASA scores (mean 2.7 vs. 2.0, p<.0001) and Charlson Comorbidity Index (mean 3.5 vs. 1.6, p<.0001), suggesting greater comorbidity burden among patients who required hospitalization. The mean proposed OAFS was also higher in inpatients than outpatients (12.4 vs. 9.2, p<.0001). The mFI-5 scores in these cohorts were 0.8 in the inpatient group and 0.3 in the outpatient group (p=.0111).

**Table 1:** Demographic characteristics.

Variable	Statistic	Outpatient surgery	Inpatient stay	Total	p-value
		(*n*=54)	(*n*=30)	(*n*=84)	
Female	*N* (%)	13 (24%)	12 (40%)	25 (30%)	.1261
Age (y)	Mean (SD)	55.3 (14.9)	68.5 (15.8)	60.0 (16.4)	**.0003**
BMI (kg/m^2^)	Mean (SD)	27.3 (4.8)	28.1 (4.3)	27.6 (4.6)	.4341
ASA score	Mean (SD)	2.0 (0.6)	2.7 (0.5)	2.3 (0.6)	**<.0001**
Charlson Comorbidity Index	Mean (SD)	1.6 (1.5)	3.5 (1.9)	2.2 (1.9)	**<.0001**
OAFS	Mean (SD)	9.2 (2.8)	12.4 (2.3)	10.4 (3.0)	**<.0001**
mFI-5	Mean (SD)	0.3 (0.5)	0.8 (1.0)	0.5 (0.8)	**.0111**
Follow-up duration (d)	Mean (SD)	207 (165)	261 (167)	227 (169)	.5426

The bold values indicate statistical signficance with p-values < 0.05.

### Operative levels

Operative details are summarized in Table 2. Patients undergoing single-level surgery were more likely to be discharged on the same day (89% of outpatients vs. 63% of inpatients; p=.0052). Conversely, patients undergoing two-level surgery were more frequently observed in the inpatient group (27% of inpatients vs. 11% of outpatients). Specific levels addressed did not significantly differ between the two groups (p=.1203), with lower lumbar levels (L3–S1) being the most common levels addressed.

**Table 2:** Operative levels.

Variable		Outpatient surgery	Inpatient stay	Total	p-value
		(*n*=54)	(*n*=30)	(*n*=84)	
Number of levels*	1 level	48 (89%)	19 (63%)	67 (80%)	**.0052**
	2 levels	6 (11%)	11 (27%)	17 (20%)	
Levels addressed*	L1–L2	1 (2%)	1 (2%)	2 (2%)	.1203
	L2–L3	5 (8%)	3 (7%)	8 (8%)	
	L3–L4	8 (13%)	12 (29%)	20 (20%)	
	L4–L5	29 (48%)	21 (51%)	50 (50%)	
	L5–S1	17 (28%)	4 (13%)	21 (21%)	

The bold values indicate statistical signficance with p-values < 0.05.

⁎ Total number of levels addressed for outpatients and inpatients are 60 and 41 respectively. A total of 101 levels were addressed among all patients.

### Surgical variables

Few meaningful differences were found in surgical variables between outpatients and inpatients (Table 3). Outpatients had a shorter mean surgical duration (113 vs. 147 minutes, p=.0010). Estimated blood loss was comparable between the groups (mean 1.9 vs. 4.1 mL, p=.1383). All patients received drains postoperatively, with inpatient stays associated with longer drain duration (mean 1.1 vs. 0.1 days for outpatients, p<.0001) and greater total drain output (mean 103 vs. 41 mL, p=.0002).

**Table 3:** Surgical variables.

Variable	Statistic	Outpatient surgery	Inpatient stay	Total	p-value
		(*n*=54)	(*n*=30)	(*n*=84)	
Length of stay (d)	Mean (SD)	0.0 (0.0)	1.5 (0.9)	0.5 (0.9)	**<.0001**
Surgical duration (min)	Mean (SD)	113 (37)	147 (54)	125 (47)	**.0010**
Estimated blood loss (mL)	Mean (SD)	1.9 (5.6)	4.1 (7.2)	2.7 (6.3)	.1383
Drain utilization	*N* (%)	54 (100%)	30 (100%)	84 (100%)	1.0000
Drain duration (d)	Mean (SD)	0.1 (0.3)	1.1 (0)	0.5 (0.6)	**<.0001**
Total drain output (mL)	Mean (SD)	41 (45)	103 (102)	64 (77)	**.0002**

The bold values indicate statistical signficance with p-values < 0.05.

### Complications

Complications were infrequent and largely similar across the cohorts (Table 4). Postoperative radiculitis occurred in 19% of outpatients and 17% of inpatients (p=.8318). Other complications, including postoperative weakness, wound complications, and reherniation, were rare and did not differ significantly between groups.

**Table 4:** Complications.

Complication	Outpatient surgery	Inpatient stay	Total	p-value
	(*n*=54)	(*n*=30)	(*n*=84)	
Postoperative radiculitis	10 (19%)	5 (17%)	15 (18%)	.8318
Postoperative weakness	1 (2%)	1 (3%)	2 (2%)	.6696
Wound complication	0 (0%)	1 (3%)	1 (1%)	.1771
Reherniation	1 (2%)	1 (3%)	2 (2%)	.6696

### Fragility scoring and predictive analysis

**Fig. 3:**
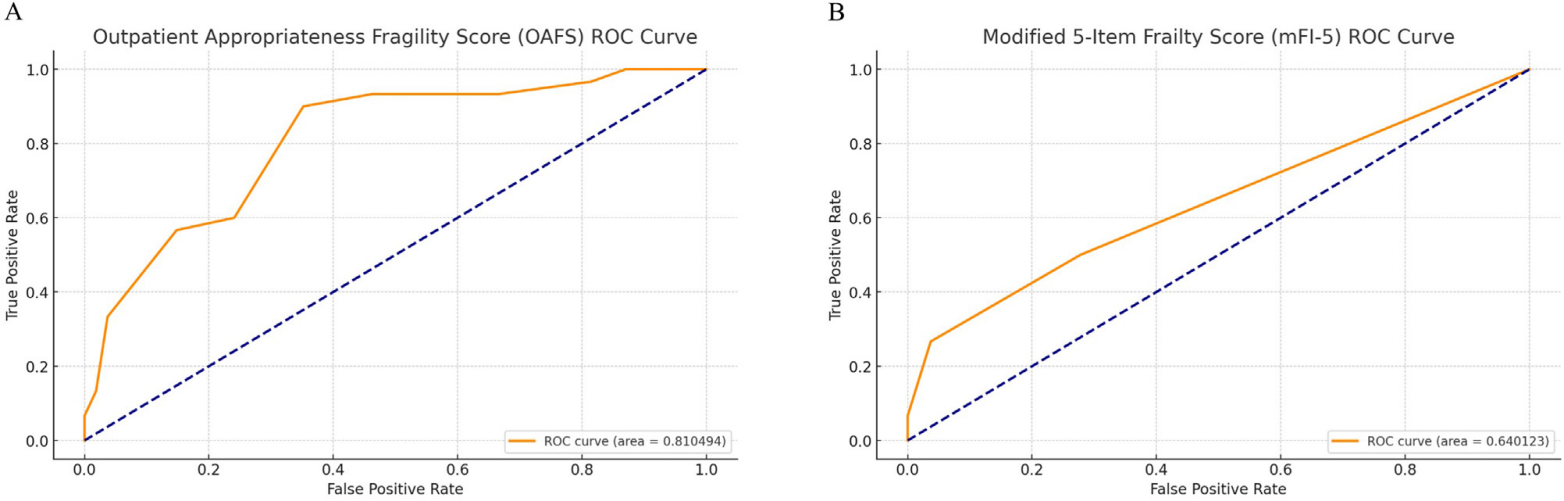
*Receiver operating characteristic (ROC) curve for the predictive value of the OAFS and mFI-5 on inpatient stay requirement*. The ROC curve illustrates the sensitivity and specificity of the OAFS (A) and mFI-5 (B) in predicting the need for inpatient stay following biportal lumbar endoscopic decompression. Using a cutoff score of ≥11, the area under the curve (AUC) for OAFS was 0.810, whereas the AUC for mFI-5 ≥ 2 was 0.640.

**Fig. 4:**
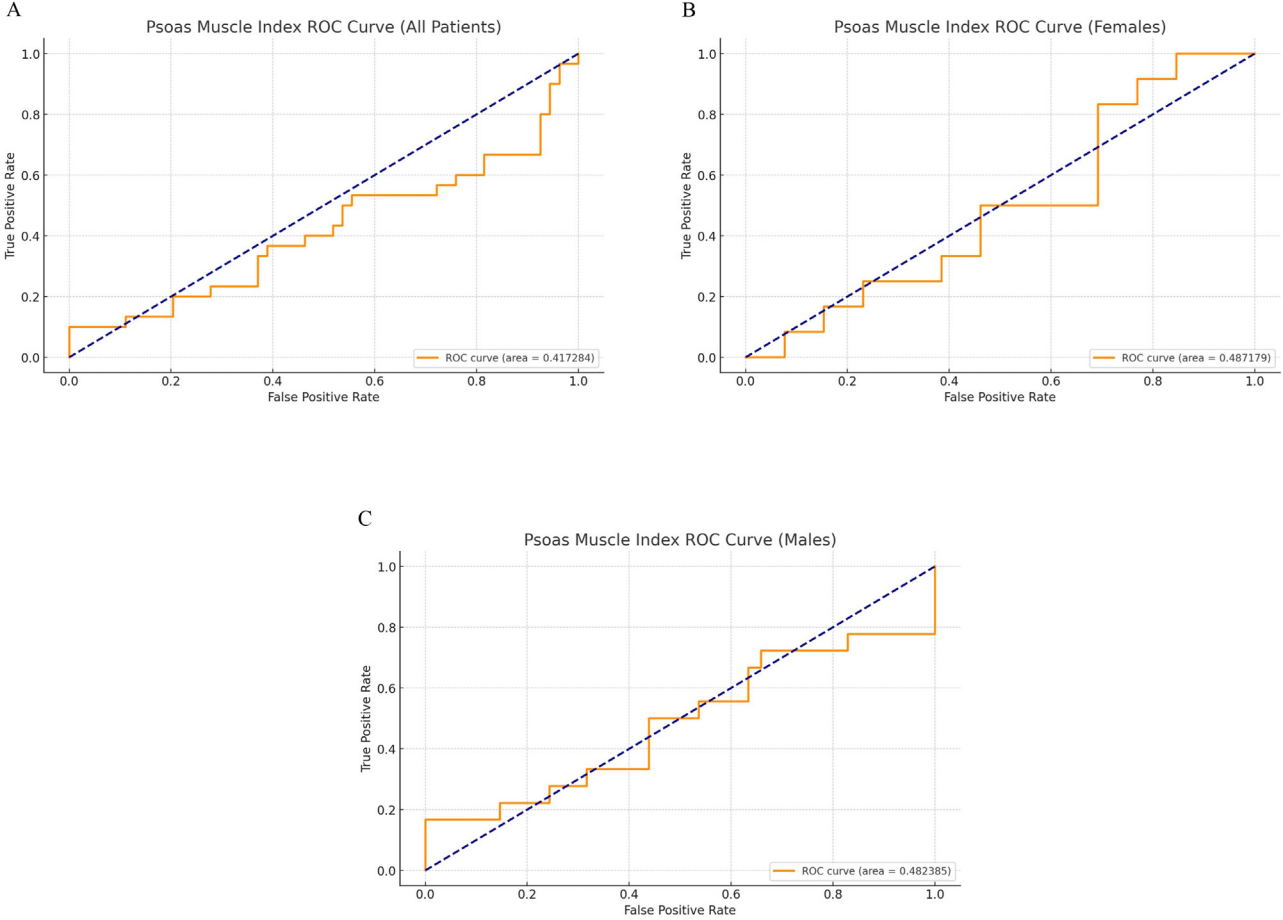
*Receiver operating characteristic (ROC) curves for psoas muscle index (PMI) as a predictor of inpatient stay following lumbar endoscopic decompression.* Part (A) represents the ROC curve for PMI across all patients, with an area under the curve (AUC) of 0.417, indicating limited predictive value for inpatient stay in the general cohort. Parts (B and C) show the ROC curve for PMI in male and female cohorts, respectively, with an AUC of 0.482 and 0.487, suggesting no significant correlation between PMI and hospital stay duration in these subgroups.

The OAFS scoring system, based on age, comorbidity burden, and type of procedure, showed predictive utility for inpatient stays. Using a cutoff value of ≥11, the score achieved an area under the curve (AUC) of 0.810, indicating a significant ability to predict the need for an inpatient stay (Fig. 3A). Similar analysis of the mFI-5 using a cutoff value of ≥2 yielded an AUC of 0.640 (Fig. 3B). The correlation between these scores is moderately positive at *r*=0.660. Evaluation of sarcopenia, represented by the PMI, revealed no significant difference between groups (p=.6732) and a low correlation with OAFS score (*r*=−0.130) and low predictive value (AUC 0.4173, Fig. 4A). Subgroup analysis by gender also showed no predictive value of PMI with an AUC of 0.482 for males (Fig. 4B) and 0.487 for females (Fig. 4C).

## Discussion

Our study introduces the OAFS, a novel fragility scoring system, to predict inpatient stay likelihood postoperatively, specifically for patients undergoing biportal lumbar endoscopic decompression. This system factors in age, comorbidities, and surgical type, reflecting an approach aligned with emerging evidence on patient stratification and risk assessment in spinal surgery. Our results corroborate findings from previous studies highlighting frailty as a determinant of postoperative outcomes in spine surgery. For instance, the modified frailty index (mFI) and other frailty scoring models have demonstrated the importance of assessing patient resilience and vulnerability to postoperative stressors [11,12]. Studies by Chotai et al. and Agarwal et al. have shown that frailty is associated with extended hospital stays, increased complication rates, and readmissions following spine surgery [9,12]. Our findings similarly underscore the role of frailty, with higher scores indicating a greater likelihood of hospitalization following procedures that are increasingly favored for outpatient settings in younger and healthier populations.

In particular, our study shows that inpatients tend to be older and present with higher ASA and Charlson Comorbidity Index scores compared to outpatients. These observations align with the evidence that age-related comorbidities complicate recovery, making frailty an essential parameter in patient selection. By integrating these factors, our OAFS score offers a practical tool for preoperative evaluation and could be instrumental in guiding clinical decisions for MISS and endoscopic decompression candidates.

The relationship between surgical approach, comorbidities, and outcomes is well-documented in MISS literature. For example, studies by Shinn et al. and Yolcu et al. have emphasized that MISS, including endoscopic decompression, is generally favorable for reducing recovery time and postoperative complications compared to open surgery, even in elderly populations [13,27]. However, complex health profiles can still lead to variable outcomes in MISS. Our analysis demonstrates that higher fragility scores are associated with inpatient stays, suggesting that age and comorbidities remain significant even with minimally invasive techniques.

Notably, we found that sarcopenia, as assessed by the PMI, did not significantly predict hospital stay. This contrasts with some studies suggesting that low muscle mass are predictors of poorer outcomes [[28], [29], [30]]. However, PMI’s low predictive value in our cohort, as indicated by an AUC of 0.417, suggests that sarcopenia may not be as critical in predicting extended stays in lumbar endoscopic decompression. This finding points to a potential limitation in using sarcopenia measures alone in risk stratification with endoscopic decompression, highlighting the value of a composite fragility score.

The novel OAFS presented here, validated with a cutoff of ≥11 points, yielded an AUC of 0.810, showing good predictive value for inpatient recovery needs. This was significantly stronger than a similar analysis of the mFI-5, which demonstrated an AUC of 0.640 and is generally below the accepted standard for a predictive test reported by Carter et al. [31]. Based on these criteria, the OAFS represents a “strong” predictive test, whereas the mFI-5 is not useful in predicting inpatient stay. This predictive capacity supports the OAFS use as an adjunctive measure in preoperative planning for lumbar decompression candidates. Given the receiver operating characteristic analysis results, the score can aid clinicians in identifying patients who may benefit from additional perioperative care resources or alternate interventions better suited to their fragility level. In this way, our scoring system aligns with the goal of individualized patient management.

### Limitations and future directions

This study’s findings have important implications for refining patient selection criteria in lumbar decompression. However, the retrospective nature of this study contributes inherent bias that limits the conclusions of this study. The study sample size is small in both cohorts, which contributes to the weaknesses of the study. The individual contribution of the OAFS components, including age, ASA, BMI, type of surgery, and mFI-5, to determine the need for inpatient stay was not investigated in this study, although each are known risk factors. Further research should explore integrating additional biomarkers or functional assessments into the OAFS to enhance its predictive accuracy. Additionally, future studies could validate the score across broader demographics and surgical settings, ensuring its generalizability and utility in diverse clinical populations.

## Conclusions

Our study demonstrates that the OAFS is a viable predictive tool for inpatient stay following endoscopic lumbar decompression, complementing existing evidence on frailty’s role in spine surgery outcomes. By enhancing preoperative risk stratification, this scoring system supports precision in patient selection, ultimately aiming to improve recovery trajectories and optimize resource allocation in minimally invasive spinal procedures.

## Summary

This study introduces the Outpatient Appropriateness Fragility Score (OAFS), a novel index of patient and surgical variables predicting inpatient stay after biportal lumbar endoscopic decompression that outperforms the modified 5-item frailty score (mFI-5).

## Example Tweet

A novel fragility score (OAFS) outperforms mFI-5 in predicting inpatient stay after biportal lumbar decompression.

## Funding

There was no financial support for this study.

## Declarations of Competing Interests

The authors declare that they have no known competing financial interests or personal relationships that could have appeared to influence the work reported in this article.

## Appendix Supplementary materials

Image, application 1
