# Supplementary material for: The utilization of a novel Outpatient Appropriateness Fragility Score to predict inpatient stay following biportal lumbar endoscopic decompression
Source: N Am Spine Soc J. 2025 Jun 18;23:100752. doi: 10.1016/j.xnsj.2025.100752 (PMC12284479; doi:10.1016/j.xnsj.2025.100752)
Supplement: Supplementary file 1 [file mmc1.docx]

# Outpatient Appropriateness Fragility Score

## Age

| Score (1-5) | Age |
| --- | --- |
| 1 | <50 |
| 2 | 50-60 |
| 3 | 60-70 |
| 4 | 70-80 |
| 5 | >80 |
|  |  |

## ASA Classification

| Score (1-5) | Grade |
| --- | --- |
| 1 | ASA 1 |
| 2 | ASA 2 |
| 3 | ASA 3 |
| 4 | ASA 4 |
| 5 | ASA 5 |

## BMI Classification (WHO)

| Score (1-5) | BMI |
| --- | --- |
| 1 | Normal weight (18.5–24.9) |
| 2 | Pre-obesity (25.0–29.9) |
| 3 | Obesity class I (30.0–34.9) |
| 4 | Obesity class II (35.0–39.9) |
| 5 | Obesity class III (Above 40) |

## Type of Surgery

| Score (1-3) | Description |
| --- | --- |
| 1 | Lumbar discectomy |
| 2 | Lumbar decompression for stenosis |
| 3 | Lumbar fusion |

## Number of Levels

| Score (1-3) | Description |
| --- | --- |
| 1 | Single level |
| 2 | Short segment (2-4 levels) |
| 3 | Long segment (>4 levels) |

## Modified Frailty Index (5-item)

| Cumulative Score (0-5) | Description |
| --- | --- |
| 1 | Diabetes mellitus |
| 1 | Congestive heart failure |
| 1 | Hypertension requiring treatment |
| 1 | Chronic obstructive pulmonary disease (COPD) or pneumonia |
| 1 | Non-independent functional health status before surgery |

Supplemental Table 1: Outpatient Appropriateness Fragility Scoring system (OAFS) incorporating age, BMI, lumbar surgery type, number of levels, and the Modified Frailty Index (5-item), range 5 to 21 points.
